# Myocardial Mitochondrial and Contractile Function Are Preserved in Mice Lacking Adiponectin

**DOI:** 10.1371/journal.pone.0119416

**Published:** 2015-03-18

**Authors:** Martin Braun, Niko Hettinger, Christoph Koentges, Katharina Pfeil, Maria C. Cimolai, Michael M. Hoffmann, Moritz Osterholt, Torsten Doenst, Christoph Bode, Heiko Bugger

**Affiliations:** 1 Division of Cardiology and Angiology I, Heart Center Freiburg University, Freiburg, Germany; 2 Department of Cardiothoracic Surgery, Jena University Hospital, Jena, Germany; 3 Institute for Clinical Chemistry and Laboratory Medicine, Freiburg University Hospital, Freiburg, Germany; Université catholique de Louvain, BELGIUM

## Abstract

Adiponectin deficiency leads to increased myocardial infarct size following ischemia reperfusion and to exaggerated cardiac hypertrophy following pressure overload, entities that are causally linked to mitochondrial dysfunction. In skeletal muscle, lack of adiponectin results in impaired mitochondrial function. Thus, it was our objective to investigate whether adiponectin deficiency impairs mitochondrial energetics in the heart. At 8 weeks of age, heart weight-to-body weight ratios were not different between adiponectin knockout (ADQ^-/-^) mice and wildtypes (WT). In isolated working hearts, cardiac output, aortic developed pressure and cardiac power were preserved in ADQ^-/-^ mice. Rates of fatty acid oxidation, glucose oxidation and glycolysis were unchanged between groups. While myocardial oxygen consumption was slightly reduced (-24%) in ADQ^-/-^ mice in isolated working hearts, rates of maximal ADP-stimulated mitochondrial oxygen consumption and ATP synthesis in saponin-permeabilized cardiac fibers were preserved in ADQ^-/-^ mice with glutamate, pyruvate or palmitoyl-carnitine as a substrate. In addition, enzymatic activity of respiratory complexes I and II was unchanged between groups. Phosphorylation of AMP-activated protein kinase and SIRT1 activity were not decreased, expression and acetylation of PGC-1α were unchanged, and mitochondrial content of OXPHOS subunits was not decreased in ADQ^-/-^ mice. Finally, increasing energy demands due to prolonged subcutaneous infusion of isoproterenol did not differentially affect cardiac contractility or mitochondrial function in ADQ^-/-^ mice compared to WT. Thus, mitochondrial and contractile function are preserved in hearts of mice lacking adiponectin, suggesting that adiponectin may be expendable in the regulation of mitochondrial energetics and contractile function in the heart under non-pathological conditions.

## Introduction

Adiponectin is an adipose tissue-derived cytokine which is abundantly present in human plasma [1]. Adiponectin exerts pleiotropic effects on its target tissues, including regulation of cellular energy metabolism. Adiponectin is able to increase glucose uptake and fatty acid oxidation in skeletal muscle, and to suppress glucose production and to decrease lipid content in the liver [2–4]. In mice, adiponectin deficiency is associated with insulin resistance, low mitochondrial content and reduced mitochondrial enzyme activity in skeletal muscle [5]. Conversely, adiponectin treatment of human myotubes in primary culture induces mitochondrial biogenesis, palmitate oxidation, and citrate synthase activity [5]. Finally, Iwabu and colleagues demonstrated that adiponectin is capable of activating peroxisome proliferator-activated receptor γ coactivator 1α (PGC-1α) signaling and mitochondrial biogenesis by activating Ca^2+^/calmodulin-dependent protein kinase kinase b (CaMKKb), or by activating the AMP-activated protein kinase (AMPK)/sirtuin 1 (SIRT1) signaling cascade in skeletal muscle [6]. Thus, adiponectin regulates cellular energy metabolism, including mitochondrial biogenesis and energetics.

The heart is highly dependent on continuous ATP delivery from mitochondrial substrate oxidation to main cardiac pump function. Impairment in mitochondrial function is observed in numerous cardiac pathologies, including the diabetic and the failing heart [7,8]. Mitochondrial dysfunction is also a major determinant of myocardial injury following ischemia-reperfusion [9]. Serum adiponectin levels are lower in patients with clinical manifestations of coronary artery disease, and adiponectin deficiency in mice leads to increased myocardial infarct size following ischemia reperfusion [10,11]. A decrease in serum adiponectin levels is associated with progression of left ventricular hypertrophy with diastolic dysfunction, and adiponectin deficiency in mice exaggerates cardiac hypertrophy following pressure overload [12,13]. While the mechanisms of increased infarct size or increased hypertrophy in adiponectin deficiency have been related to AMPK- and cyclooxygenase-2 (COX-2)-dependent mechanisms or activation of extracellular-signal regulated kinase (ERK), respectively, a possible contribution of mitochondrial dysfunction has not been investigated in detail to date. We hypothesize that adiponectin deficiency may impair myocardial mitochondrial function, which may potentially promote adverse cardiac outcomes following ischemia-reperfusion or pressure overload. Thus, it was our objective to investigate whether adiponectin deficiency impairs mitochondrial function in the heart.

## Methods

### Animals

Male adiponectin knockout mice (ADQ^-/-^) and respective C57BL/6J wildtype (WT) littermate controls were purchased from The Jackson Laboratories (Bar Harbor, ME) and studied at the age of 8 weeks. Animals were housed at 22°C with a 12-hour light/12-hour dark cycle with free access to water and standard chow. The study conforms to the *Guide for the Care and Use of Laboratory Animals* published by the US National Institutes of Health and was performed after securing approval by the Regierungspräsidium Freiburg.

### Isolated working heart perfusion

Hearts were excised and placed into ice-cold Krebs-Henseleit Buffer (KHB) containing (in mmol/l) 128 NaCl, 5 KCl, 1 KH_2_PO_4_, 1.3 MgSO_4_, 15 NaHCO_3_, 2.5 CaCl_2_ and 5 Glucose. Non-cardiac tissue was removed and the aorta was cannulated and secured to a 20-gauge plastic cannula. Following retrograde Langendorff perfusion (50 mmHg perfusion pressure) with KHB at 37°C, the left atrium was cannulated using an 18-gauge metal cannula, and perfusion was switched to working mode with 15 mmHg preload and 50 mmHg afterload. After an initial equilibration period, hearts were perfused for 60 min with KHB containing 0.4 mmol/l palmitate bound to 3% BSA. Aortic pressure changes were measured using a pressure catheter placed inside the aortic cannula (Millar Micro-Tip, Millar Instruments, Houston, TX). Aortic developed pressure, cardiac output and cardiac power were quantified to evaluate contractile function. Myocardial oxygen consumption (MVO_2_) was measured as difference of percent oxygen concentration in pre- and postcardial buffer samples using a fiber-optic oxygen sensor (Ocean Optics, Orlando, FL). Cardiac efficiency was calculated as ratio of hydraulic work to MVO_2_. Glucose oxidation and glycolytic flux were determined in the same perfusion using [U-^14^C] glucose and [5–^3^H] glucose (each specific activity 300 MBq/mol). Glucose oxidation was determined by the sum of trapped ^14^CO_2_ in hyamine hydroxide and the presence of ^14^C bicarbonate anion in the perfusate released from [U-^14^C] glucose. Glycolytic flux was determined by measuring the amount of ^3^H_2_O released from the metabolism of [5–^3^H] glucose by separating H_2_O from the perfusate using an anion exchange resin (200–400 mesh Dowex AG-1-X4) column technique. Palmitate oxidation was measured in separately perfused hearts by determining the amount of ^3^H_2_O released from [9,10–^3^H] palmitate (specific activity, 500 GBq/mol). For details, see S1 Methods.

### Myocardial triacylglycerol content

Triacylglycerol levels were quantified enzymatically in whole heart homogenates using the Serum Triglyceride Determination Kit (Sigma Aldrich, Taufkirchen, Germany).

### Isoproterenol treatment

Mice were anesthetized with ketamin (100 mg/kg) and xylazin (40 mg/kg), and mini-osmotic pumps (250 μl; Alzet, Cupertino, CA) were implanted subcutaneously in the intrascapular area. Pumps were filled under sterile conditions in accordance with manufacturer's instructions, containing 50 μl 0.2% ascorbic acid and either body weigh-adjusted amounts of isoproterenol (17mg/kg/day) or saline solution. Pumps had a mean flow rate of 0.25 μl/h for 5 days before harvesting the hearts.

### OXPHOS complex activities

Mitochondria were isolated by differential centrifugation as described before [18]. Protein concentration of mitochondrial suspensions was determined using the Micro BCA method (Pierce, Rockford, IL). Samples of fresh mitochondria were treated with 10 mg cholate/mg mitochondrial protein and diluted to a final concentration of 1 mg/ml with MSM/EDTA buffer (5 mmol/l mannitol, 220 mmol/l sucrose, 5 mmol/l MOPS, 2 mmol/l EDTA, pH 7.4) supplemented with 1 μl/ml mammalian protease inhibitor cocktail (Sigma Aldrich, Taufkirchen, Germany). Complex I activity was measured based on a modified protocol by Janssen et al. [14]. 30 μl of isolated mitochondria at a concentration of 0.1 g/l was added to 150 μl incubation buffer (200 mmol/l KH_2_PO_4_, 14,7 mmol/l BSA, 1.3 mmol/l 2,6-dichloroindophenol (DCIP), 10 mmol/l decylubichinon, 0.11 mmol/l antimycin A). After 3 minutes of incubation, 20 μl 2.1 mmol/l NADH was added, and the reduction of DCIP was followed spectrophotometrically at 600 nm. After 60 s, 10 μl of 0.15 mmol/l rotenone was added to inhibit complex I activity. Complex II activity was measured according to Krahenbuhl et al. [15] with modifications. 45 μl of 0,5 g/l isolated mitochondria were added to 525 μl incubation buffer (in mmol/l: KH_2_PO_4_ 50, DCIP 0.05, antimycin A 0.005, duroquinone 0.5, EDTA 1; 1 g/l BSA) for 3 minutes. 60 μl of 210 mmol/l succinate was added, and the reduction of DCIP was followed spectrophotometrically at 600 nm. Cytochrome c oxidase activity was measured according to Wharton et al. [16]. Mitochondria (0.005 g/l) were measured in 175 μl incubation buffer (50 mmol/l KH_2_PO_4_, 0.04 mmol/l dodecyl-β-D-maltoside). 30 μl of 0.28 mmol/l reduced cytochrome c was added and oxidized by complex IV, followed spectrophotometrically at 550 nm.

### Mitochondrial respiration and ATP synthesis

Saponin-permeabilized cardiac fibers were prepared from freshly excised hearts as described previously [17,18]. Respiration rates of fibers were measured in a buffer (25°C) containing (in mmol/l) KCL 125, HEPES 20, Mg-Acetate 3, EGTA 0.4, BSA 2mg/ml, KH_2_PO_4_ 5, DTT 0.3, glutamate 5, malate 2. Respiration was measured in the presence of substrate alone (V_0_), after addition of 1 mmol/l ADP (V_ADP_), and after addition of 1 μg/ml oligomycin (V_Oligo_). To determine rates of ATP synthesis, separate experiments were performed with identical buffer conditions. 0.6 mmol/l ADP was added, and samples (10 μl) were taken every 10 s for a total period of 1 min and added to 190 μl DMSO. ATP content of ATP-containing DMSO samples was determined using a bioluminescence assay kit (Enliten Luciferase/Luciferin Reagent; Promega, Mannheim, Germany).

### Immunoprecipitation

Whole hearts were homogenized in 500 μl ice-cold lysis buffer (25 mmol/L Tris HCl, ph 7.4, 5 mmol/L MgCl_2_, 10% glycerol, 100 mmol/L KCl, 1% NP40, 0.3 mmol/L dithiothreitol, 5μl protease/phosphatase inhibitor cocktail, 5 mmol/L nicotinamide, 1 μmol/L orthovanadate, 50 mmol/L sodium fluoride, 1 mmol/L sodium butyrate and 5 mmol/L sodium pyrophosphate), sonicated for 1 min using an Ultra-Turrax T10 basic (IKA Labortechnik, Staufen, Germany), and separated by centrifugation (10 000 x g for 30 min at 4°C). 500 μg of protein were incubated with 50 μl beads (1.5 mg) and 5 μg anti-PGC-1α antibody (Santa Cruz, Heidelberg, Germany), rotating overnight at 4°C. Beads were collected using a magnetic rack, washed with washing-buffer (Immunoprecipitation kit, Invitrogen, Carlsbad, CA), dissolved in loading buffer, and boiled. The immunoprecipitates were then separated by SDS-PAGE and immunoblotted using anti-PGC-1α-antibody (1:200; Santa Cruz, Heidelberg, Germany) and subsequently with acetyl-lysine antibody (1:1.000; Cell Signaling, Boston, MA).

### Western blot analysis

Proteins of whole heart homogenates or isolated mitochondria were separated by SDS-PAGE and transferred onto PVDF membranes (Bio-Rad Laboratories, Hercules, CA), probed with primary antibodies overnight at 4°C, washed with PBS, and probed with secondary antibodies for 1 h at room temperature. The following primary antibodies were used: Anti-AMPK 1:500 (# 2532, Cell Signaling, Boston, MA), Anti-phospho-AMPK Thr 172 1:500 (#2531, Cell signaling, Boston, MA), Anti-complex I NDUFB8 subunit 1:2000 (#459210, Invitrogen, Carlsbad, CA), Anti-complex II Fp subunit 1:10000 (#MS204, Mitosciences, Eugene, OR), Anti-complex IV subunit IV 1:1000 (#MS407, Mitosciences, Eugene, OR), Anti-CTRP9 (#A00081-03-100, Aviscera Bioscience, Santa Clara, CA). Secondary antibodies: Anti-Mouse IgG Fab2 Alexa Fluor, Anti-Rabbit IgG (H+L) Fab2, Alexa Fluor 1:1000 (#4410, #4414, both Cell Signaling, Boston, MA). Detection and quantification of fluoresence bands was performed using the Odyssey imaging system (LI-COR Biosciences, Lincoln, NE). Loading control was performed using staining with Coomassie Blue R-250 (Bio-Rad, Hercules,CA).

### Gene expression

Total RNA was isolated from hearts with TRIzol reagent (Invitrogen, Carlsbad,CA), purified with the RNEasy Kit (Qiagen, Hilden, Germany), and reverse transcribed using the SuperScriptIII Reverse Transcriptase Kit (Invitrogen, Carlsbad, CA), as described before [17]. SYBR-green (Invitrogen, Carlsbad, CA) was used as probe, and amplification was monitored using the CF X96 Real-Time PCR system (Bio Rad, Munich, DE). Data were normalized by expressing them relative to the levels of the invariant transcript 16S ribosomal RNA and are presented as arbitrary units normalized to wildtype expression levels. Primer sequences and accession numbers are presented in S1 Table.

### SIRT1 activity

Freshly excised hearts were homogenized in buffer containing 250 mmol/L sucrose, 10 mmol/L Tris-HCl, and 1 mmol/L EDTA (adjusted to pH 7.4) using a Polytron homogenizer. The homogenate was frozen (liquid N_2_) and thawed (37°C) three times, and SIRT1 activity was measured using the SIRT1 Drug Discovery Kit (Biomol, Hamburg, Germany). In brief, 0.5 mg/ml homogenate protein were incubated in 50 μl Biomol assay buffer containing 0.05 mmol/L *Fluor de Lys* SIRT1 substrate and 5μmol/L NAD^+^ ± 2.5 mmol/L suramin, incubated at 37°C for 45 min, and fluorophore generation equivalent to deacetylated SIRT1 substrate levels was measured 15 min following addition of 50 μl 1mmol/L nicotinamide and Developer II solution in a fluorescence plate reader (Excitation: 360nm, Emission: 460nm).

### Electron microscopy

Left ventricular samples were fixed in 2.5% glutaraldehyde and 1% paraformaldehyde, postfixed in 2% osmium, embedded in resin, and sectioned (thickness 80–100 nm). Mitochondrial morphology was assessed at x 2,000 and x 40,000 magnifications. Mitochondrial volume density was analyzed by stereology in a blinded fashion using the pointcounting method [17,19].

### Statistical analysis

Data are presented as means ± SE. When comparing two groups, significance was determined using a Student’s t test. Data generated following isoproterenol treatment were analyzed using a 2-way analysis of variance (2-way ANOVA) followed by a Bonferroni post-hoc test. For all analyses, GraphPad Prism software was used (GraphPad Software, Inc., La Jolla, CA), and significant difference was accepted when p < 0.05.

## Results

### Preserved mitochondrial and contractile function in hearts of ADQ^-/-^ mice

Male animals were investigated at 8 weeks of age. Serum adiponectin levels were 24 μg/ml in WT mice, which is consistent with other reports [1,20]. As expected, serum adiponectin was litterally not detectable in ADQ^-/-^ mice (S1 Fig.). In glucose tolerance tests, ADQ^-/-^ mice showed no difference in glucose clearance compared to WT mice (S1 Fig.). Serum levels of free fatty acids and triglycerides were significantly lower in ADQ^-/-^ mice (S1 Fig.). While heart weights and body weights were mildly increased in ADQ^-/-^ mice, heart weight-to-body weight ratios were not different compared to WT mice (S2 Table).

**Fig 1:**
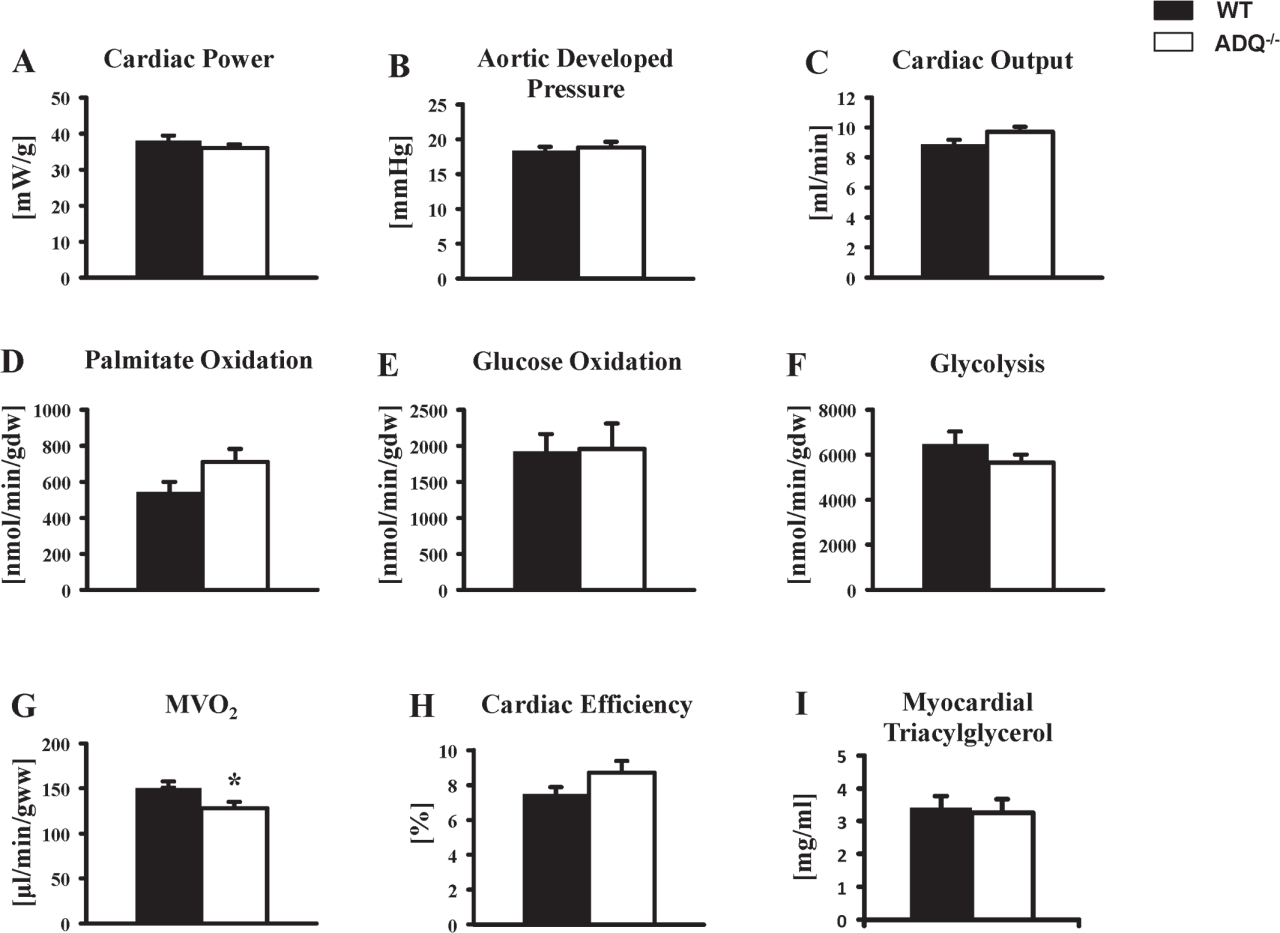
Preserved contractile function in ADQ^-/-^ hearts. Cardiac power (A), aortic developed pressure (B), cardiac output (C), palmitate oxidation (D), glucose oxidation (E), glycolysis (F), MVO_2_ (G), and cardiac efficiency (H) in isolated working hearts of ADQ^-/-^ and WT mice at 8 weeks of age; n = 4–6 for substrate oxidation, n = 10 for contractile parameters. (I) Myocardial triacylglycerol levels in ADQ^-/-^ and WT mice at 8 weeks of age; n = 4–5. * p<0.05 vs. WT.

ADQ^-/-^ and WT mice were perfused in the isolated working heart mode. Cardiac power, aortic developed pressure and cardiac output were all unchanged between groups (Fig. 1). Cardiac power was also unchanged between ADQ^-/-^ and WT mice when analyzing contractile function separately in perfusions measuring fatty acid oxidation or glucose oxidation, although cardiac power was higher both in WT and ADQ^-/-^ mice in the glucose oxidation group compared to the fatty acid oxidation group (S2 Fig.). Rates of palmitate oxidation, glucose oxidation and glycolysis were also not different between groups (Fig. 1). When relating substrate oxidation rates to cardiac work, palmitate and glucose oxidation rates were also not different between groups, although glycoclysis rates were now reduced in ADQ^-/-^ mice compared to WT mice (S3 Fig.). A mild but significant decrease in MVO_2_ in ADQ^-/-^ mice did not result in a relevant increase in cardiac efficiency (Fig. 1). Myocardial triacylglycerol levels were unchanged between groups (Fig. 1). Thus, cardiac contractile function and energy substrate utilization rates were mainly unaffected in the absence of adiponectin.

**Fig 2:**
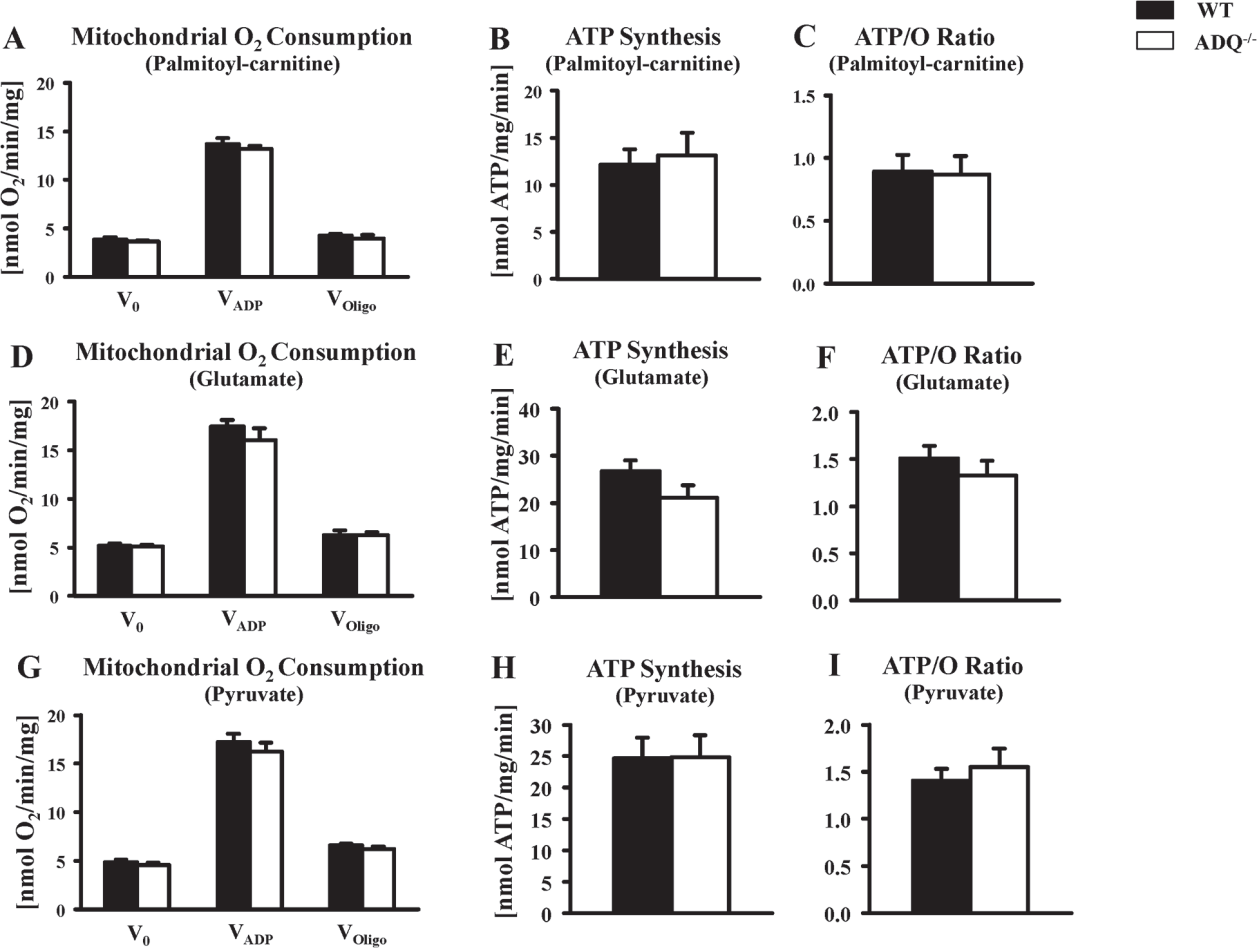
Preserved mitochondrial function in ADQ^-/-^ hearts. Mitochondrial O_2_ consumption rates, ATP synthesis rates, and ATP/O ratios in saponin-permeabilized cardiac fibers of ADQ^-/-^ and WT mice, using palmitoyl-carnitine (A-C), glutamate (D-F), or pyruvate (G-I) as substrate; n = 6.

**Fig 3:**
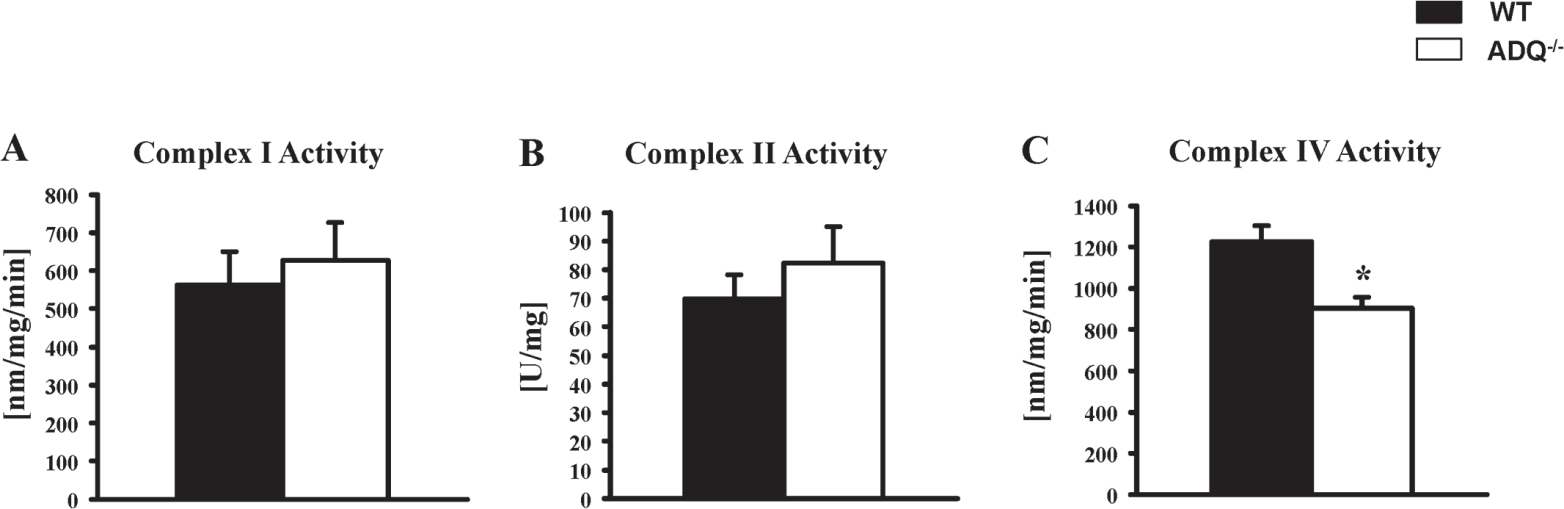
Preserved mitochondrial OXPHOS complex activities in ADQ^-/-^ hearts. Enzymatic activity of complex I subunit NDUFB8 (A), complex II subunit 30kDa (B), and complex IV subunit II (C) in isolated mitochondria of ADQ^-/-^ and WT hearts; n = 5–6. * p<0.05 vs. WT.

**Fig 4:**
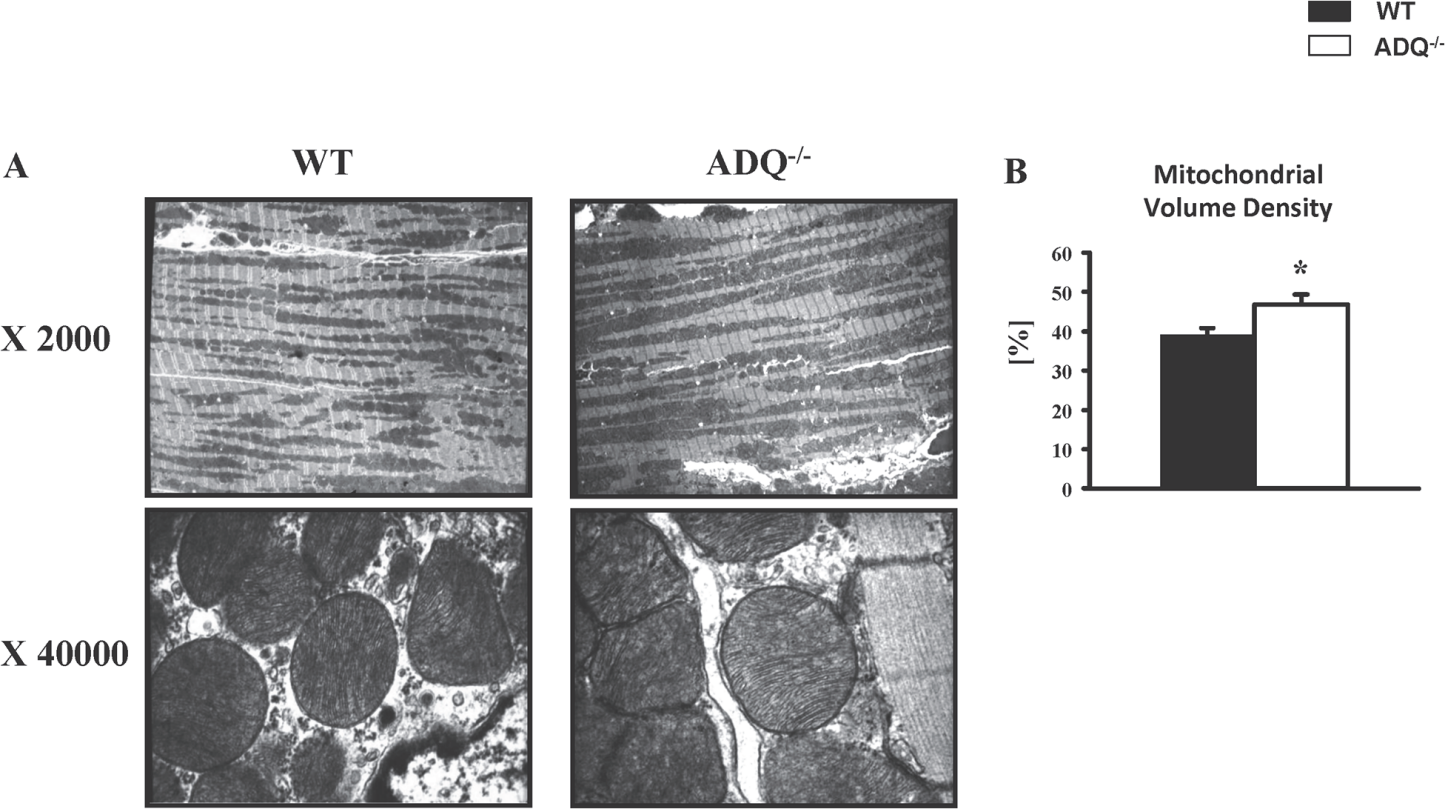
Preserved mitochondrial morphology in ADQ^-/-^ hearts. Representative electron microscopy images at magnification x 2000 or x 40000 (A), and stereologic quantification of mitochondrial volume density (B) of 8 week-old ADQ^-/-^ and WT hearts; n = 4. * p<0.05 vs. WT.

Mitochondrial function was evaluated in saponin-permeabilized cardiac fibers. V_0_, V_ADP_ and V_Oligo_ were unchanged between ADQ^-/-^ and WT mice, using palmitoyl-carnitine, glutamate or pyruvate as substrate (Fig. 2). Similarly, rates of ATP synthesis were unchanged between groups with any substrate, resulting in unchanged ATP/O ratios (Fig. 2). In addition, we determined enzymatic activities of OXPHOS complexes. While complex IV showed a significant decrease in enzymatic activity, activities of complexes I and II were unchanged (Fig. 3). Mitochondrial ultrastructure was analyzed using electron microscopy. Mitochondria were embedded in regular fashion between myofibers without disarrays, and cristae density and membrane morphology appeared identical in ADQ^-/-^ and WT mice (Fig. 4). Mitochondrial volume density was even slightly increased in ADQ^-/-^ mice (Fig. 4). Taken together, mitochondrial function was preserved in hearts of ADQ^-/-^ mice.

**Fig 5:**
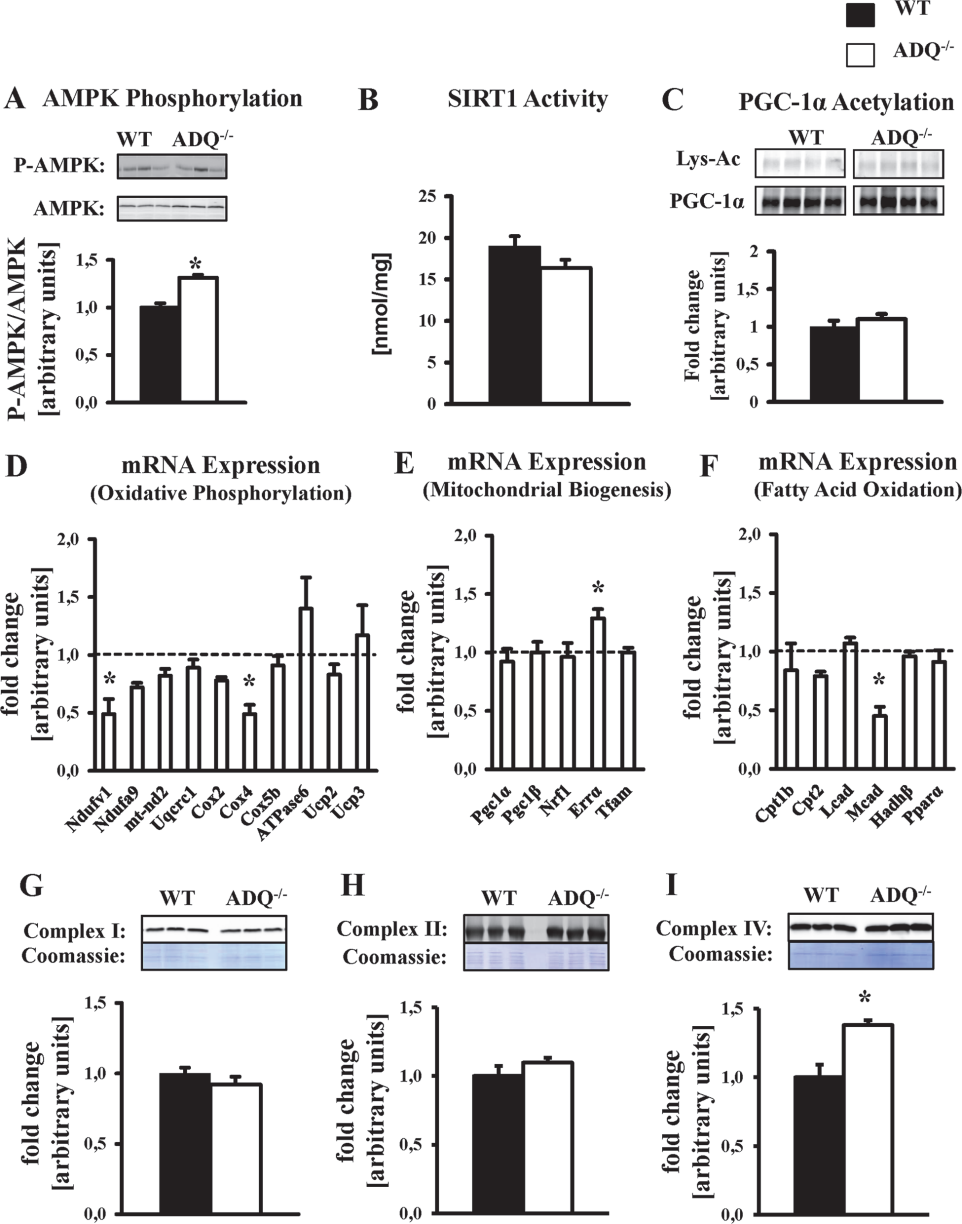
Preserved OXPHOS protein levels and mitochondrial biogenic signaling in ADQ^-/-^ hearts. AMPK phosphorylation (A), SIRT1 activity (B), lysine acetylation (Lys-Ac) of PGC-1α (C), mRNA expression of OXPHOS subunits (D), mRNA expression of mitochondrial biogenesis signaling molecules (E), mRNA expression of fatty acid oxidation genes and PPARα (F), and mitochondrial protein levels of OXPHOS complexes I (NDUFB8 subunit; G), II (Fp subunit; H), and IV (subunit IV; I) in hearts of ADQ^-/-^ and WT mice at 8 weeks of age; n = 4–5. Myocardial mRNA expression is expressed relative to WT expression which was set to 1 (indicated by the dotted line). * p<0.05 vs. WT.

In skeletal muscle, lack of adiponectin impairs AMPK phosphorylation and SIRT1 activity and thereby signaling via PGC-1α, resulting in decreased expression of OXPHOS genes [6]. In ADQ^-/-^ hearts, AMPK phosphorylation was not decreased but even increased compared to WT hearts (Fig. 5). Both SIRT1 activity and lysine acetylation of PGC-1α were not different between groups (Fig. 5). While mRNA expression of two OXPHOS subunits and of medium chain acyl-CoA dehydrogenase (Mcad) was reduced in ADQ^-/-^ hearts, mRNA expression of six other OXPHOS subunits was unchanged (Fig. 5). On the protein level, mitochondrial content of OXPHOS complexes I (subunit NDUFB8), II (subunit 30kDa) and IV (subunit II) was not reduced or even increased in ADQ^-/-^ mice (Fig. 5). In addition, mRNA expression of transcription factors and cofactors involved in mitochondrial biogenesis was maintained, including PGC-1α, and ERRα mRNA expression was even increased in ADQ^-/-^ mice (Fig. 5). Thus, lack of adiponectin did not impair mitochondrial biogenic signaling in the heart.

### Preserved mitochondrial and contractile function in hearts of ADQ^-/-^ mice in response to prolonged isoproterenol stimulation

**Fig 6:**
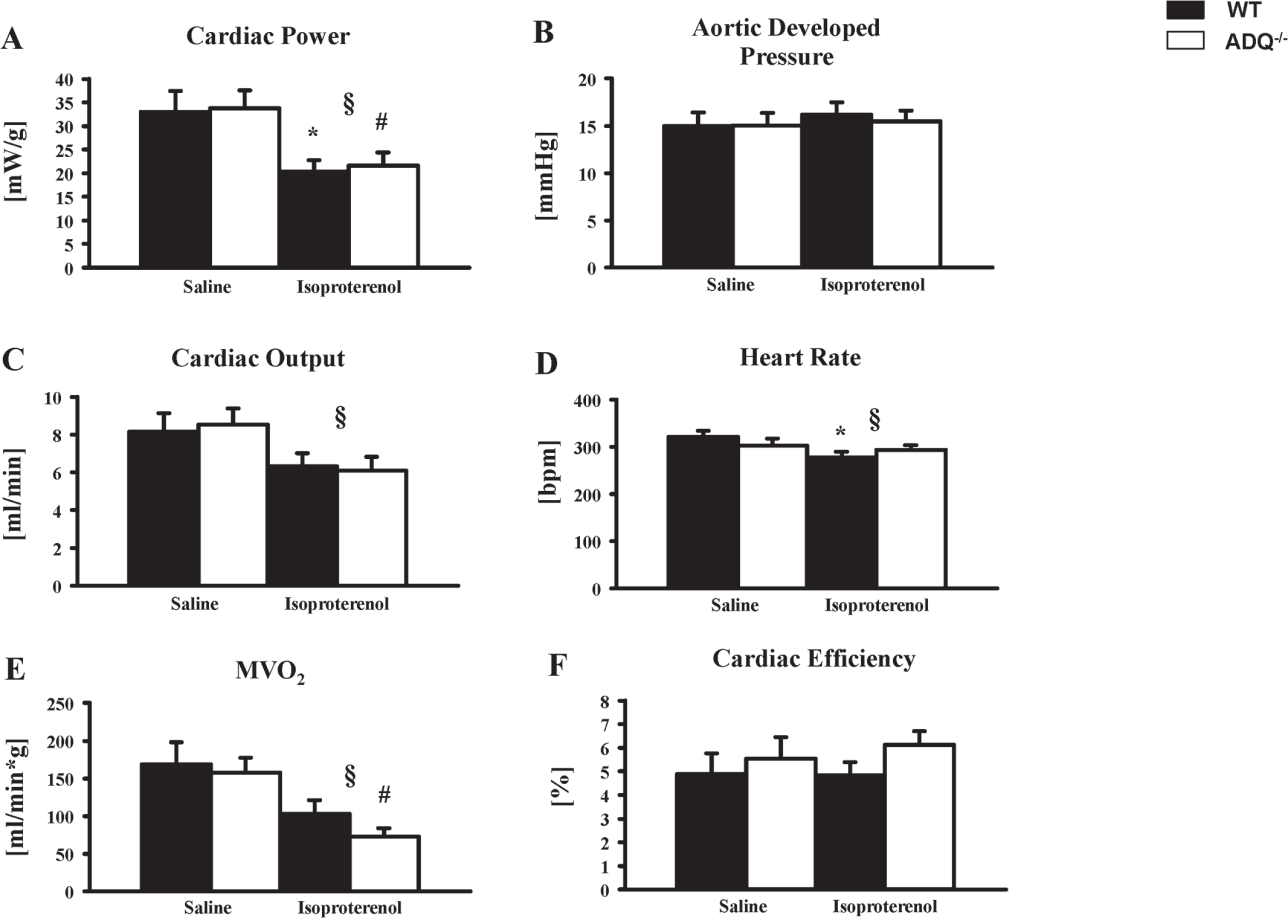
Similar contractile response of ADQ^-/-^ hearts to isoproterenol stimulation. Heart weight-to-tibia length ratios (A), and aortic developed pressure (B), cardiac output (C), cardiac power (D), MVO_2_ (E), and cardiac efficiency (F) in isolated working hearts of ADQ^-/-^ and WT mice subjected to 5 days of continuous subcutaneous isoproterenol infusion; n = 4. 2-way ANOVA: § effect of isoproterenol, * p<0.05 vs. WT saline, # p<0.05 vs. ADQ^-/-^ saline.

To investigate the response to a prolonged increase in energy demand, mice were subjected to prolonged beta-adrenergic stimulation by continuous subcutaneous infusion of isoproterenol for 5 days using mini-osmotic pumps. Isoproterenol infusion resulted in increased heart weight-to-body weight ratios both in WT and ADQ^-/-^ mice compared to saline-treated mice, but no difference was observed between isoproterenol-treated WT and ADQ^-/-^ mice (S3 Table). While aortic developed pressure remained unchanged among groups in isolated working hearts following isoproterenol infusion, cardiac output and cardiac power were reduced in isoproterenol-treated mice compared to saline-treated mice, likely due to a decrease in heart rate in the isoproterenol-treated groups (Fig. 6). Importantly, no differences in contractile function were observed between ADQ^-/-^ and WT mice following isoproterenol treatment. MVO_2_ showed an additional decrease in ADQ^-/-^ mice compared to WT mice following isoproterenol treatment, which however did not result in a significant increase in cardiac efficiency in isoproterenol-treated ADQ^-/-^ mice (Fig. 6). Thus, the contractile response following prolonged beta-adrenergic stimulation was similar between ADQ^-/-^ and WT mice.

**Fig 7:**
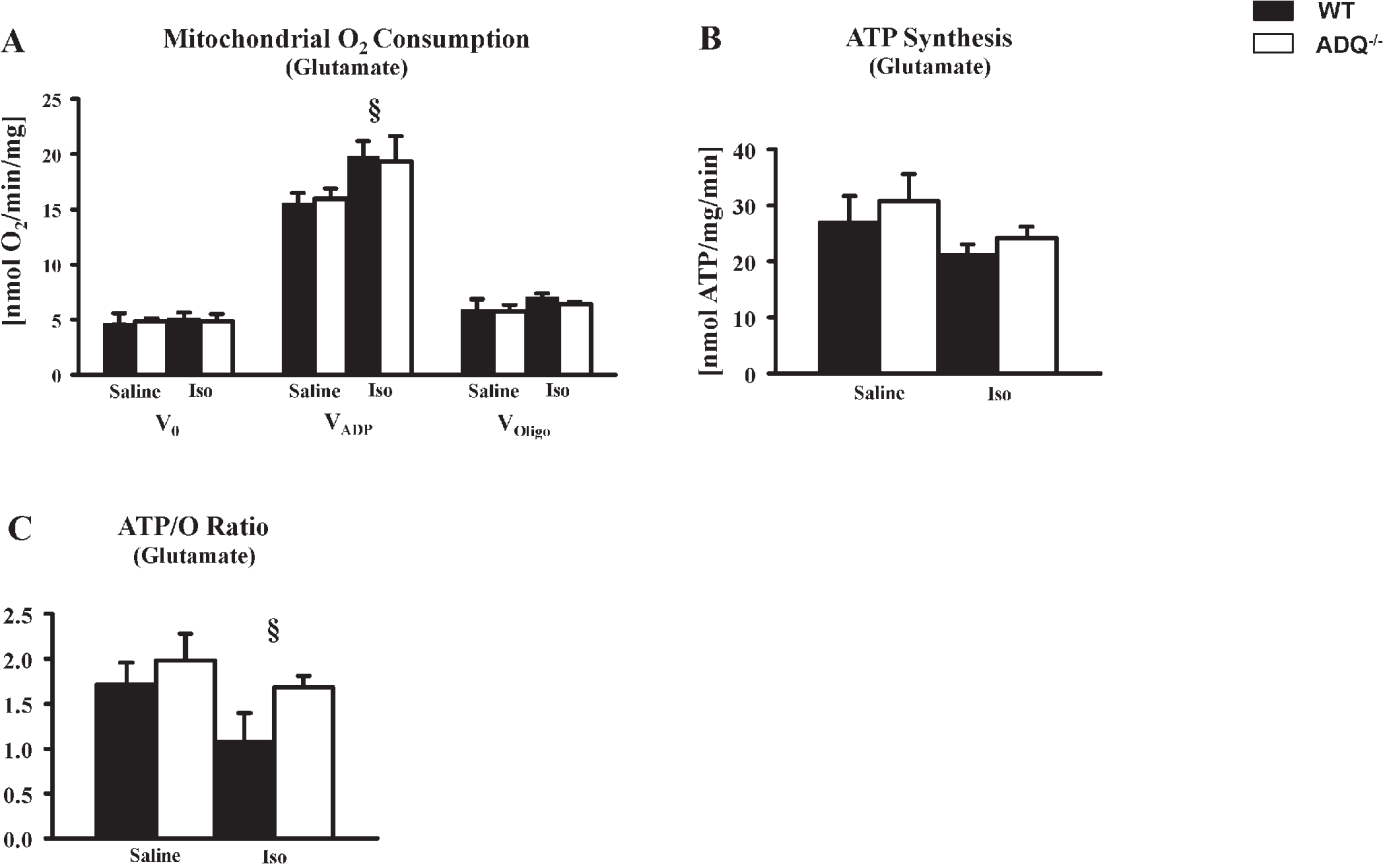
Preserved mitochondrial function in ADQ^-/-^ hearts following isoproterenol treatment. Mitochondrial O_2_ consumption rates (A), ATP synthesis rates (B), and ATP/O ratios (C) in saponin-permeabilized cardiac fibers of ADQ^-/-^ and WT mice using glutamate as substrate; n = 4. 2-way ANOVA: § effect of isoproterenol.

Mitochondrial function was investigated following prolonged isoproterenol stimulation. In saponin-permeabilized cardiac fibers, V_ADP_ was increased in ADQ^-/-^ and WT mice following isoproterenol treatment compared to saline-treated mice (Fig. 7). However, V_ADP_ was not different between ADQ^-/-^ and WT mice. Similarly, no changes in ATP synthesis were observed between genotypes following isoproterenol treatment (Fig. 7). ATP/O ratios were decreased following isoproterenol treatment compared to saline-treated mice, whereas no differences were observed between ADQ^-/-^ and WT mice following isoproterenol or saline treatment.

### Increased serum protein levels of CTRP9 in ADQ^-/-^ mice

**Fig 8:**
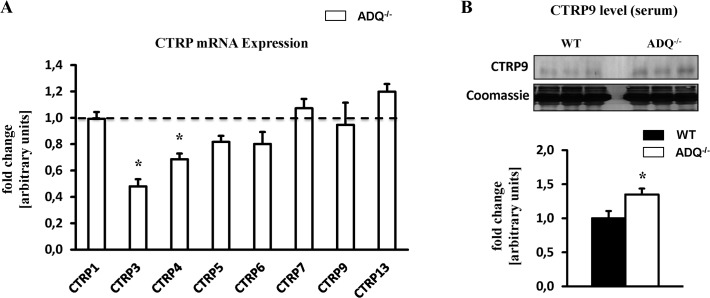
Myocardial expression of CTRPs. (A) Myocardial mRNA expression of CTRP1, 3, 4, 5, 6, 7, 9 and 13 in ADQ^-/-^ mice at 8 weeks of age, relative to WT expression which was set to 1 (indicated by the dotted line); n = 8. (B) Serum CTRP9 protein levels in ADQ^-/-^ and WT mice at 8 weeks of age; n = 5. * p<0.05 vs. WT.

C1q/TNF-related proteins (CTRPs) are recently discovered adiponectin paralogs which bind to adiponectin receptors and may mimic adiponectin effects on cellular physiology [21]. In particular, CTRP9 has been shown to increase AMPK activity and mitochondrial content in skeletal muscle, and is able to signal via adiponectin receptor 1 [27,28]. CTRPs circulate in the bloodstream and/or are expressed in cells in a tissue-specific manner. Of the 15 CTRPs identified thus far, we were able to detect the mRNA expression of 8 CTRPs in myocardial tissue. While myocardial mRNA expression of CTRP3 and CTRP4 was reduced in ADQ^-/-^ mice, the expression of CTRP1, 5, 6, 7, 9 and 13 was not different compared to WT mice (Fig. 8). In contrast, serum protein levels of CTRP9 were increased in ADQ^-/-^ mice (Fig. 8), suggesting that increased CTRP9 signaling via adiponectin receptors may compensate for the lack of adiponectin action in ADQ^-/-^ mice.

## Discussion

In the current study, we investigated mitochondrial energetics and cardiac function in mice lacking adiponectin. Mitochondrial respiratory capacity, ATP synthesis, activities of OXPHOS complexes I and II, and mitochondrial biogenic signaling were unaffected by lack of adiponectin. Similarly, myocardial contractile function was unaffected, even in response to prolonged beta-adrenergic stimulation with isoproterenol. Thus, mitochondrial and contractile function are preserved in hearts of mice lacking adiponectin, suggesting that adiponectin may be expendable in the regulation of mitochondrial energetics and contractile function in the heart under non-pathologic conditions.

We show here that mitochondrial respiratory capacity and ATP synthesis were not impaired in saponin-permeabilized cardiac fibers of AdQ^-/-^ mice. Similarly, activity of OXPHOS complexes I and II was not different between groups, and signaling via the AMPK-PGC-1α axis appeared unaffected by adiponectin deficiency. Finally, rates of oxidative substrate utilization in isolated working hearts were not impaired in AdQ^-/-^ mice. These data are in line with O`Shea et al. who reported of unaffected citrate synthase activity in adiponectin knockout mice [22,23]. In contrast, Yan et al. reported a decrease in AMPK phosphorylation, an increase in PGC-1α acetylation, and a mild decrease of the enzymatic activity of OXPHOS complexes I, III and V in ADQ^-/-^ mice, concluding that impaired mitochondrial biogenic signaling may impair mitochondrial function in mice lacking adiponectin [24]. Unfortunately, mitochondrial respiratory capacity, which includes the activity of all OXPHOS complexes and is considered as an overall measure of mitochondrial function, was not reported, thereby limiting conclusions on overall mitochondrial function. In addition, no data on gene or protein expression of OXPHOS complex subunits was presented, limiting the conclusion of impaired mitochondrial biogenesis as a mechanism contributing to impaired OXPHOS complex activity in mice lacking adiponectin. In the current study, we found no convincing evidence of impairment in AMPK-PGC-1α signaling, mitochondrial respiration, ATP synthesis, and gene and protein expression of OXPHOS subunits. Thus, we would argue that, in this model of adiponectin deficiency, adiponectin may not be essential to maintain mitochondrial function in the heart.

Cardiac function in isolated working hearts was not impaired in AdQ^-/-^ mice under non-stressed conditions. This finding is in line with several other reports showing no impairment of cardiac function in adiponectin knockout mice using echocardiography [13,23,25], suggesting that adiponectin deficiency may not be required to maintain physiological cardiac function under non-stressed conditions. Following prolonged beta-adrenergic stimulation with isoproterenol for 5 days, both contractile and mitochondrial function were not differentially affected in ADQ^-/-^ mice compared to WT mice. Thus, even when increasing energy demand for prolonged time periods, adiponectin deficiency does not seem to predispose for myocardial energy depletion. When extrapolating these results, one may argue that impaired energetics may also not contribute to exaggerated hypertrophy following pressure overload, although the adaptive mechanisms in response to increased energy demand due to aortic constriction are quite different compared to increased beta-adrenergic stimulation as occurs in the isoproterenol model. Ultimately, myocardial energetics need to be investigated in adiponectin-deficient mice following aortic constriction to evaluate a contribution of impaired energetics to exaggerated hypertrophy.

When integrating both the data on mitochondrial function from Yan et al. [24] and our study, one needs to conclude that adiponectin deficiency in the mouse model generated by Chan and colleagues [26] only mildly impairs myocardial mitochondrial function at the most. It can however not be ruled out that other members of the C1q/TNF-related protein (CTRP) family with high amino acid sequence homology to adiponectin may compensate for the lack of adiponectin action by mimicking adiponectin action and signaling. CTRPs circulate in the bloodstream and/or are expressed in cells in a tissue-specific manner. First, we investigated myocardial mRNA expression of all detectable CTRPs, which revealed decreased mRNA expression for CTRP3 and CTRP4, and unchanged expression for the remaining detectable CTRPs in ADQ^-/-^ mice. CTRP3 replenishment following myocardial infarction has been shown to improve survival rates, to restore cardiac function, to attenuate cardiomyocyte apoptosis, to reduce fibrosis, and to increase revascularization, thus suggesting that reduced myocardial CTRP3 expression rather does not result in compensatory effects in the absence of adiponectin [29]. The functional effects of CTRP4 are largely unknown, particularly in the heart. In contrast, when looking at circulating CTRPs, we found increased serum protein levels of CTRP9 in ADQ^-/-^ mice (Fig. 8B). Since overexpression of CTRP9, the closest paralog of adiponectin, has been shown to increase AMPK activity and mitochondrial content in skeletal muscle, and since CTRP9 is able to signal via adiponectin receptor 1 [27,28], increased CTRP9 signaling via adiponectin receptor 1 may compensate for the lack of adiponectin action in ADQ^-/-^ mice. We did not investigate serum levels of other CTRPs or the expression of CTRPs in extramyocardial tissues in ADQ^-/-^ mice, which needs to be addressed in future studies in this animal model. Finally, studies using mice with deficiency of adiponectin receptors may allow a more differentiated view on the role of adiponectin signaling in myocardial energetics and may circumvent potential confounding effects by CTRPs.

It remains to be mentioned that adiponectin deficiency has been shown to impair mitochondrial function and biogenesis in extracardiac tissues such as skeletal muscle. Civitarese and colleagues showed that mitochondrial DNA copy number, cytochrome c oxidase activity and citrate synthase activity were impaired in skeletal muscle of adiponectin-deficient mice generated by Philipp Scherer`s group [5, 30]. In addition, Iwabu and colleagues demonstrated that lack of adiponectin receptor 1 in skeletal muscle also impairs mitochondrial DNA content, PGC-1α signaling and mitochondrial F_O_F_1_-ATPase activity [6]. It remains to be elucidated whether tissue-specific differences in adiponectin action and/or compensatory mechanisms in our animal model may be responsible for the different effects of adiponectin deficiency on myocardial mitochondrial biology. Compensatory mechanisms may include increased CTRP9 action on myocardial adiponectin receptors (Fig. 8B), or adaptive posttranslational mechanisms as suggested by increased mitochondrial Cox2 protein levels despite unchanged Cox2 mRNA expression (Fig. 5D, I).

In conclusion, our data suggest that, at least in the model used in this study, adiponectin is not required to maintain mitochondrial energetics and contractile function in the heart under non-pathological conditions. A possible compensating effect due to increased CTRP9 action, which may mimic cellular adiponectin effects, cannot be ruled out. Analysis of adiponectin receptor signaling and downstream effects in the myocardium will help to further elucidate the relevance of myocardial adiponectin action for cardiac energetics and for myocardial adaptations in response to pathologic stressors.

## Supporting Information

S1 Fig Glucose tolerance, serum adiponectin and serum metabolite levels. Serum adiponectin levels (A), glucose tolerance test (B), and serum levels of free fatty acids (C) and triglycerides (D) in ADQ^-/-^ and WT mice at 8 weeks of age; n = 5–6. * p<0.05 vs. WT.
(TIF)

S2 Fig Cardiac power in different working heart perfusion groups. Cardiac power calculated separately for ADQ^-/-^ and WT mice used to measure palmitate oxidation or used to measure glucose oxidation; n = 4–6. * p<0.05.
(TIFF)

S3 Fig Substrate oxidation normalized to cardiac work. Rates of palmitate oxidation (A), glycolysis (B) and glucose oxidation (C) normalized to cardiac work in ADQ^-/-^ and WT mice.
(TIFF)

S1 Methods Detailed description of serum metabolite measurements, glucose tolerance tests and isolated working heart experiments. (DOCX)

S1 Table Sequences of forward and reverse primers used for RT-PCR. Abbreviations: ATPase6, ATP synthase F0 subunit 6; Cox II, cytochrome c oxidase subunit II; Cox IV, cytochrome c oxidase subunit IV; Cox Vb, cytochrome c oxidase subunit Vb; Cpt1b, carntine palmitoyltransferase 1b; Cpt2, carnitine palmitoyltransferase 2; CTRP, C1q/TNF-related protein; Errα, estrogen related receptor alpha; Hadhβ, hydroxyacyl-CoA dehydrogenase, β subunit; Lcad, long chain acyl-CoA dehydrogenase; mt-Nd2, NADH dehydrogenase 2, mitochondrial; Mcad, medium chain acyl-CoA dehydrogenase; Ndufv1, NADH dehydrogenase [ubiquinone] flavoprotein 1; Ndufa9, NADH dehydrogenase [ubiquinone] 1 alpha subcomplex subunit 9; Uqcrc1, ubiquinol cytochrome c reductase core protein 1; Nrf1, nuclear respiratory factor 1; Pgc-1α, peroxisome proliferator-activated receptor gamma coactivator 1 alpha; Pgc-1β; peroxisome proliferator-activated receptor gamma coactivator 1 beta; PPARα, peroxisome proliferator-activated receptor α; Tfam, mitochondrial transcription factor A; Ucp2, uncoupling protein 2; Ucp3, uncoupling protein 3.
(DOCX)

S2 Table Similar heart weight-to-body weight ratio in ADQ^-/-^ mice. Heart weights related to body weight in ADQ^-/-^ and WT mice at 8 weeks of age; n = 10. * p<0.05 vs. WT.
(DOCX)

S3 Table Effect of isoproterenol treatment on heart weight-to-body weight ratio in WT and ADQ^-/-^ mice. Heart weights, body weights and heart weight-to-body weight (HW-BW) ratios in ADQ^-/-^ and WT mice following isoproterenol or saline treatment; n = 7. 2-way ANOVA: § effect of isoproterenol, $ effect of genotype. * p<0.05 vs. WT saline, # p<0.05 vs. ADQ^-/-^ saline.
(DOCX)
