# Supplementary figures and images for: Myocardial Mitochondrial and Contractile Function Are Preserved in Mice Lacking Adiponectin
Source: PLoS One. 2015 Mar 18;10(3):e0119416. doi: 10.1371/journal.pone.0119416 (PMC4364743; doi:10.1371/journal.pone.0119416)

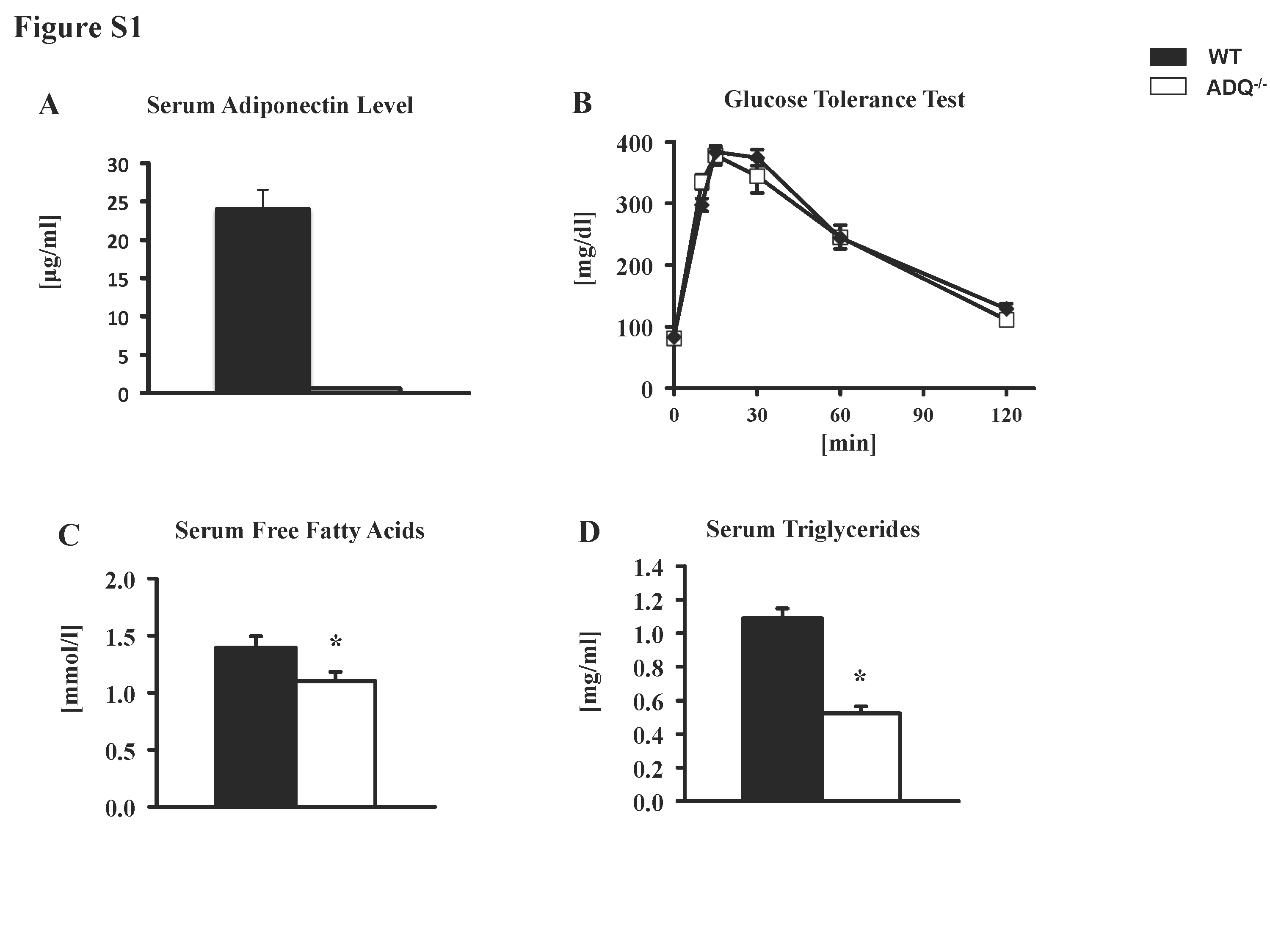

Supplement: S1 Fig — Serum adiponectin levels (A), glucose tolerance test (B), and serum levels of free fatty acids (C) and triglycerides (D) in ADQ-/- and WT mice at 8 weeks of age; n = 5–6. * p<0.05 vs. WT. (TIF) [file pone.0119416.s001.tif]
