# Supplementary material for: Myocardial Mitochondrial and Contractile Function Are Preserved in Mice Lacking Adiponectin
Source: PLoS One. 2015 Mar 18;10(3):e0119416. doi: 10.1371/journal.pone.0119416 (PMC4364743; doi:10.1371/journal.pone.0119416)
Supplement: S1 Methods — (DOCX) [file pone.0119416.s004.docx]

**S1 Methods**

*Glucose tolerance tests:* Mice underwent an intraperitoneal injection of glucose (100 mg/ml) after 12 h of starvation. Blood glucose levels were measured using a standard glucometer.

*Serum fatty acid and triglyceride levels:* Serum free fatty acids were measured using the free fatty acids half micro test kit (Roche Diagnostics, Indianapolis, IN). Serum triglycerides were quantified enzymatically using the Serum Triglyceride Determination Kit (Sigma Aldrich, Taufkirchen, Germany).

*Isolated working heart perfusion:* Animals were weighed and anesthetized using 0.3 mg/g body weight thiopenthal i.p.. Before excising the heart, mice were heparinized with an intracaval injection of 200 I.U. heparin. Hearts were excised together with lungs and placed into Krebs-Henseleit Buffer (KHB) at room temperature containing (in mmol/L) 128 NaCl, 5 KCl, 1 KH_2_PO_4_, 1.3 MgSO_4_, 15 NaHCO_3_, 2.5 CaCl_2_ and 5 Glucose. After removing the lungs, hearts were placed into ice cold KHB for further preparation. Other non-cardiac tissue was removed and the aorta was canulated and bound to a 20-gauge plastic canula. Perfusion was carried out using water jacketed glass jars warmed to 38.5 °C resulting in a final myocardial perfusion temperature of approx. 37 °C. Hearts were initially perfused in a retrograde Langendorff mode (50 mmHg perfusion pressure) with KHB solution gassed with 95% O_2_ and 5% CO_2_ resulting in pH 7.4. Subsequently, the left atrium was canulated within 60 s using a 18-gauge metal cannula and sealed using 4/0 surgical suture. After switching to the working mode, hearts were perfused for 60 min with 15 mmHg preload and 50 mmHg afterload using KHB with 0.4 mmol/L palmitate bound to 3 % BSA. Aortic pressure changes were measured using a pressure catheter placed inside the aortic cannula (Millar Micro-Tip, Millar Instruments, Houston, TX). Aortic developed pressure was calculated as the difference of systolic and systemic pressure. Heart rate was determined from the pressure traces by measuring the time interval between peak systolic values. In conjunction with pressure measurements, aortic and coronary flow measurements were obtained by collecting the flow from the afterload line and the effluent dripping off the heart, respectively. To accomplish these flow measurements without disrupting metabolic measurements, the perfusion apparatus was made air-tight. Graduated syringes were connected and sealed within the perfusion apparatus and flow was determined (at 20-min intervals) by measuring the time required to collect a 2 ml sample for coronary flow and 5 ml sample for aortic flow. Cardiac output (ml/min) was calculated as the sum of aortic and coronary flow. Cardiac power (mW/g) was calculated as the product of cardiac output and afterload per dry heart weight. Cardiac work (ml*mmHg/min) was calculated as product of cardiac output and aortic developed pressure per minute. Hydraulic work (J/min*g) was calculated as the product of cardiac output and aortic developed pressure per wet heart weight (WHW).

*Myocardial oxygen consumption, cardiac efficiency and substrate oxidation rates:* Myocardial oxygen consumption (MVO_2_) was measured every 20 min during working heart perfusions as the difference of percent oxygen concentration in pre- (arterial (aO_2_)) and postcardial (venous (vO_2_)) buffer samples. Oxygen concentration in the samples was measured using a fiber-optic oxygen sensor (Ocean Optics, Orlando, FL). The following formulae were used to determine MVO_2_ (μl/min/gww) and cardiac efficiency (%): MVO_2_ = (aO_2_-vO_2_) * (coronary flow/WHW) * (718/760) * (1000 * Bunsen coefficient); where Bunsen coefficient for plasma is 0.0212, and where 718 and 760 mmHg are atmospheric pressures in Freiburg and at sea level, respectively. Cardiac efficiency = hydraulic work/MVO_2_ * 100. MVO_2_ was converted to µmol/min by multiplying by the conversion factor 0.0393 and then to Joules (J/min) using the conversion of 1 µmol O_2_ = 0.4478 J as described by Suga et al. [[1](#_ENREF_1)]. Palmitate oxidation was measured in the same perfusion by determining the amount of ^3^H_2_O released from [9,10-3H] palmitate (specific activity, 500 GBq/mol). ^3^H_2_O was separated from [9,10-3H] palmitate by mixing 500 µl perfusate sample with 1.88 ml Chloroform/Methanol (1:2 v/v) (15 min incubation) followed by the addition of 625 µl chloroform (15 min incubation). Then 2 mol/L HCl/KCl solution was added, mixed and incubated for at least 30 min until a polar and a non-polar phase appeared. 1.8 ml of the polar phase was transfered into another tube and mixed with 1 ml of chloroform, 1ml of Methanol and 900 µl of HCl/KCl solution, and mixed and incubated for 15 min after each step. After the last addition and at least 30 min incubation, two 500 µl aliquots were taken from the upper layer and counted for ^3^H. Palmitate oxidation rates were calculated from ^3^H_2_O production, taking into account the dilution factor incurred from the process of separating ^3^H_2_O from [9,10-3H] palmitate. Glucose oxidation and glycolysis were measured in separately perfused hearts. Glucose oxidation was determined by trapping ^14^CO_2_ released from [U-^14^C] glucose (specific activity, 300 MBq/mol) in 15 ml of hyamine hydroxide (Fischer Scientific, Schwerte, Germany). After the addition of 4 ml UltimaGold scintillation cocktail, samples were counted for ^14^C. Since ^14^CO_2_ is also dissolved in the form of bicarbonate anion, a 3.5 ml sample was drawn every 20 min and injected under 1 ml of mineral oil to avoid CO_2_ gassing out. From this sample 750 µl were taken and injected into 750 µl 9N H_2_SO_4_ in a sealed 10 ml BD vacutainer tube capped with a hyamine hydroxide filter lined scintillation vial. Tubes were vortexed every 5 min for 1 h. In the end, the scintillation vial was disconnected from the vacutainer tube and after the addition of 5 ml UltimaGold scintilation cocktail, counted for ^14^C. Glycolytic flux was determined by measuring the amount of ^3^H_2_O released from the metabolism of [5-^3^H] glucose (specific activity 300 MBq/mol). To separate ^3^H_2_O from [5-^3^H] glucose and [U-^14^C] glucose, perfusate samples were passed through an anion exchange resin (200-400 mesh Dowex AG-1-X4) column pretreated with 1 mol/L NaOH and then converted to the borate form using 0.5 mol/L boric acid. Columns were washed 5 times, 100 µl buffer sample was loaded, and the sample was eluated with 800 µl of H_2_O. After addition of 3 ml UltimaGold scintillation cocktail samples were counted for ^3^H and ^14^C. The amount of ^3^H_2_O measurements were corrected for the column efficiency retaining glucose by measuring the amount of [U-^14^C] glucose which passed the column. This signal was equaled to the amount of [5-^3^H] glucose which also passes the column and was accounted to contribute to the ^3^H signal.

**References:**

1. Suga H. Ventricular energetics. Physiol Rev. 1990;70: 247-277.
