# Supplementary material for: Myocardial Mitochondrial and Contractile Function Are Preserved in Mice Lacking Adiponectin
Source: PLoS One. 2015 Mar 18;10(3):e0119416. doi: 10.1371/journal.pone.0119416 (PMC4364743; doi:10.1371/journal.pone.0119416)
Supplement: S1 Table — Abbreviations: ATPase6, ATP synthase F0 subunit 6; Cox II, cytochrome c oxidase subunit II; Cox IV, cytochrome c oxidase subunit IV; Cox Vb, cytochrome c oxidase subunit Vb; Cpt1b, carntine palmitoyltransferase 1b; Cpt2, carnitine palmitoyltransferase 2; CTRP, C1q/TNF-related protein; Errα, estrogen related receptor alpha; Hadhβ, hydroxyacyl-CoA dehydrogenase, β subunit; Lcad, long chain acyl-CoA dehydrogenase; mt-Nd2, NADH dehydrogenase 2, mitochondrial; Mcad, medium chain acyl-CoA dehydrogenase; Ndufv1, NADH dehydrogenase [ubiquinone] flavoprotein 1; Ndufa9, NADH dehydrogenase [ubiquinone] 1 alpha subcomplex subunit 9; Uqcrc1, ubiquinol cytochrome c reductase core protein 1; Nrf1, nuclear respiratory factor 1; Pgc-1α, peroxisome proliferator-activated receptor gamma coactivator 1 alpha; Pgc-1β; peroxisome proliferator-activated receptor gamma coactivator 1 beta; PPARα, peroxisome proliferator-activated receptor α; Tfam, mitochondrial transcription factor A; Ucp2, uncoupling protein 2; Ucp3, uncoupling protein 3. (DOCX) [file pone.0119416.s005.docx]

**S1 Table. Sequences of forward and reverse primers used for RT-PCR.**

**Gene Primer Primer Sequence (5´-3`)**

α1-Tubulin Forward AAGGAGGATGCTGCCAATAA

Reverse AGGTGAGCCAGAGCCAGT

ATPase6 Forward CAAACAAATAATGCTAATCCACACACC

Reverse GCTGTAAGCCGGACTGCTAATG

Cox II Forward CCATCCCAGGCCGACTAA

Reverse CAGAGCATTGGCCATAGAATAACC

Cox IV Forward CGCTGAAGGAGAAGGAGAAG

Reverse GCAGTGAAGCCAATGAAGAA

Cox Vb Forward GGAAGTGCATCTGCTTGTCTC

Reverse TAGGGACACCACCTCCAGAA

Cpt1b Forward TGCCTTTACATCGTCTCCAA

Reverse AGACCCCGTAGCCATCATC

Cpt2 Forward CCAGCTGACCAAAGAAGCA

Reverse GCAGCCTATCCAGTCATCGT

CTRP1 Forward TCCGAGCTCTGTTGACATGC

Reverse AAAGATTGACCAGCCCCTGG

CTRP3 Forward CATCTGGTGGCACCTGCTG

Reverse TGACACAGGCAAAATGGGAG

CTRP4 Forward TTAGCCACGATCACGATGGCT

Reverse TGTACTTGCCGTGGTTGCTGT

CTRP5 Forward TGGAGTCTGAGCCTCCG

Reverse AGAAGGGCAAGAAGTGGCCT

CTRP6 Forward ATCACAGACATGGGCCAAGG

Reverse TCAACTCACAGACCCCGGAC

CTRP7 Forward GACGAGTCTTGCCATCTGTGC

Reverse TTAGCCTGATTGGCCCGAG

CTRP9 Reverse TGGTGAACGTGGTGCCTACA

Forward TGCAGTCACATCCCACCCT

CTRP13 Forward AACGCAAGATAAGCAGATGTGTG

Reverse AAGGAGTATTTGCTTTGGCGG

Errα Forward GGAGGACGGCAGAAGTACAA

Reverse CAGGTTCAACAACCAGCAGA

Hadhβ Forward GCCAACAGACTGAGGAAGGAA

Reverse ACACTGGCAAGGCTGGATT

Lcad Forward ATGGCAAAATACTGGGCATC

Reverse TCTTGCGATCAGCTCTTTCA

Mcad Forward ACTGACGCCGTTCAGATTTT

Reverse GCTTAGTTACACGAGGGTGATG

Mt-Nd2 Forward CGCCCCATTCCACTTCTGATTACC

Reverse TTAAGTCCTCCTCATGCCCCTATG

Ndufv9 Forward ATCCCTTACCCTTTGCCACT

Reverse CCGTAGCACCTCAATGGACT

Ndufv1 Forward TGTGAGACCGTGCTAATGGA

Reverse CATCTCCCTTCACAAATCGG

Uqcrc1 Forward TGCCAGAGTTTCCAGACCTT

Reverse CCAAATGAGACACCAAAGCA

Nrf1 Forward CTTCAGAACTGCCAACCACA

Reverse GCTTCTGCCAGTGATGCTAC

Pgc-1α Forward GTAAATCTGCGGGATGATGG

Reverse AGCAGGGTCAAAATCGTCTG

Pgc-1β Forward TGAGGTGTTCGGTGAGATTG

Reverse CCATAGCTCAGGTGGAAGGA

PPARα Forward GAGAATCCACGAAGCCTACC

Reverse AATCGGACCTCTGCCTCTTT

Tfam Forward CAAAAAGACCTCGTTCAGCA

Reverse CTTCAGCCATCTGCTCTTCC

Ucp2 Forward TCTCCTGAAAGCCAACCTCA

Reverse CTACGTTCCAGGATCCCAAG

Ucp3 Forward tttggagctggctctgtg

Reverse aaggccctcttcagttgctc

Abbreviations: ATPase6, ATP synthase F0 subunit 6; Cox II, cytochrome c oxidase subunit II; Cox IV, cytochrome c oxidase subunit IV; Cox Vb, cytochrome c oxidase subunit Vb; Cpt1b, carntine palmitoyltransferase 1b ; Cpt2, carnitine palmitoyltransferase 2 ; CTRP, C1q/TNF-related protein ; Errα, estrogen related receptor alpha ; Hadhβ, hydroxyacyl-CoA dehydrogenase, β subunit; Lcad, long chain acyl-CoA dehydrogenase; mt-Nd2, NADH dehydrogenase 2, mitochondrial; Mcad, medium chain acyl-CoA dehydrogenase; Ndufv1, NADH dehydrogenase [ubiquinone] flavoprotein 1; Ndufa9, NADH dehydrogenase [ubiquinone] 1 alpha subcomplex subunit 9 ; Uqcrc1, ubiquinol cytochrome c reductase core protein 1; Nrf1, nuclear respiratory factor 1; Pgc-1α, peroxisome proliferator-activated receptor gamma coactivator 1 alpha; Pgc-1β; peroxisome proliferator-activated receptor gamma coactivator 1 beta; PPARα, peroxisome proliferator-activated receptor α ; Tfam, mitochondrial transcription factor A; Ucp2, uncoupling protein 2; Ucp3, uncoupling protein 3;
