# Supplementary material for: Myocardial Mitochondrial and Contractile Function Are Preserved in Mice Lacking Adiponectin
Source: PLoS One. 2015 Mar 18;10(3):e0119416. doi: 10.1371/journal.pone.0119416 (PMC4364743; doi:10.1371/journal.pone.0119416)
Supplement: S2 Table — Heart weights related to body weight in ADQ-/- and WT mice at 8 weeks of age; n = 10. * p<0.05 vs. WT. (DOCX) [file pone.0119416.s006.docx]

**S2 Table.** **Similar heart weight-to-body weight ratio in ADQ^-/-^ mice.**

|  | WT | | | ADQ^-/-^ | | | |  |
| --- | --- | --- | --- | --- | --- | --- | --- | --- |
| Heart weight [mg] | 110.8 | ± | 1.9 | | 120.6 | ± | 2.7 * | |
| Body weight [g] | 22.3 | ± | 0.4 | | 24.6 | ± | 0.4 * | |
| Heart weight-body weight ratio [mg/g] | 5.1 | ± | 0.1 | | 4.9 | ± | 0.1 | |

Heart weights related to body weight in ADQ^-/-^ and WT mice at 8 weeks of age; n=10.

* p<0.05 vs. WT
