# Supplementary material for: Myocardial Mitochondrial and Contractile Function Are Preserved in Mice Lacking Adiponectin
Source: PLoS One. 2015 Mar 18;10(3):e0119416. doi: 10.1371/journal.pone.0119416 (PMC4364743; doi:10.1371/journal.pone.0119416)
Supplement: S3 Table — Heart weights, body weights and heart weight-to-body weight (HW-BW) ratios in ADQ-/- and WT mice following isoproterenol or saline treatment; n = 7. 2-way ANOVA: § effect of isoproterenol, $ effect of genotype. * p<0.05 vs. WT saline, # p<0.05 vs. ADQ-/- saline. (DOCX) [file pone.0119416.s007.docx]

**S3 Table. Effect of isoproterenol treatment on heart weight-to-body weight ratio in WT and ADQ^-/-^ mice.**

|  | Saline | | | | | | | | | | |  | | | | Isoproterenol | | | | | | | | | | | |  |  |  |  |  |  |  |  |  |
| --- | --- | --- | --- | --- | --- | --- | --- | --- | --- | --- | --- | --- | --- | --- | --- | --- | --- | --- | --- | --- | --- | --- | --- | --- | --- | --- | --- | --- | --- | --- | --- | --- | --- | --- | --- | --- |
|  | | WT | | | | | ADQ^-/-^ | | | | | | |  | | | | | | | WT | | | ADQ^-/-^ | | | | | | | | | |  |  |  |
| Heart weight (mg) | | 123.6 | ± | 6.6 | | | | 136.8 | | ± | 4.8 ^$^ | | | | | |  | 154.7 | | | | ± | 6.9 * ^§^ | | | | 175.5 | | ± | | 6.7 ^# § $^ | | | | |  |
| Body weight (g) | | 23.8 | ± | 0.5 | | | | 23.9 | | ± | 0.5 | | | | | |  | 22.5 | | | | ± | 0.6 | | | | 24.1 | | ± | | 1.1 | | | | |  |
| HW-BW ratio (mg/g) | | 5.2 | ± | 0.2 | | | | 5.7 | | ± | 0.2 | | | | | |  | 6.9 | | | | ± | 0.2 * ^§^ | | | | 7.3 | | ± | | 0.3 ^# §^ | |  |  |  |  |
|  |  | | | |  |  | | |  | | | |  | |  | | | |  |  | | | | |  |  | | | |  | |  | | |  | |

Heart weights, body weights and heart weight-to-body weight (HW-BW) ratios in ADQ^-/-^ and WT mice following isoproterenol or saline treatment; n=7.

2-way ANOVA: § effect of isoproterenol, $ effect of genotype.

* p<0.05 vs. WT saline, # p<0.05 vs. ADQ^-/-^ saline.
